# Global Patterns of Fatalities With Toxicological Detection of Novel Psychoactive Substances: A Systematic Review and Meta-analysis

**DOI:** 10.7759/cureus.99586

**Published:** 2025-12-18

**Authors:** Swati Tyagi, Jaspinder Pratap Singh, Abid Manzoor, Sunny Basra, Yashpal Sharma, Palak Sharma, Shubham, Arashpreet Kaur, Sant Kaur

**Affiliations:** 1 Forensic Medicine, All India Institute of Medical Sciences, New Delhi, IND; 2 Forensic Medicine, Shri Mata Vaishno Devi Institute of Medical Excellence, Katra, IND; 3 Physiology, Shri Mata Vaishno Devi Institute of Medical Excellence, Katra, IND; 4 Forensic Medicine, Guru Gobind Singh Medical College, Faridkot, IND; 5 Hospital Administration, Shri Mata Vaishno Devi Institute of Medical Excellence, Katra, IND; 6 General Physician, Department of Health and Family Welfare, Amritsar, IND; 7 Neuropsychiatry, Bhatia Neuropsychiatric Hospital, Amritsar, IND; 8 Health and Family Welfare, Ayushman Arogya Kendra Focal Point, Jalandhar, IND; 9 Medicine, Government Medical College, Amritsar, IND

**Keywords:** cannabinoids, cathinones, drug-related mortality, forensic toxicology, novel psychoactive substances, synthetic opioids

## Abstract

Novel psychoactive substances (NPS) have rapidly diversified the global drug market, creating escalating challenges for forensic toxicology and mortality surveillance. While regional reports have described fatal NPS involvement, the global prevalence and temporal trends of NPS detection in fatalities are insufficiently quantified.

A search following the Preferred Reporting Items for Systematic reviews and Meta-Analyses (PRISMA) 2020 guidelines was performed in PubMed, Embase, Scopus, and Web of Science (January 2008-May 2025), supplemented by screening a repository of 96 primary studies, to find studies with toxicologically confirmed detection of NPS in fatalities. We included autopsy or surveillance datasets with analytical confirmation by gas chromatography-mass spectrometry, liquid chromatography-tandem mass spectrometry (LC-MS/MS), high-resolution mass spectrometry, or nuclear magnetic resonance spectroscopy. The primary outcome was the proportion of NPS-positive cases among all forensic cases undergoing comprehensive toxicological analysis. Random-effects meta-analysis estimated pooled proportions and subgroup trends. Heterogeneity, I², meta-regression by year, region, and analytical method, and publication bias by Egger's test were assessed.

Of 621 records screened (525 from databases and 96 from supplementary repository), 125 studies met the inclusion criteria and 86 contributed to quantitative synthesis. The pooled global proportion of fatalities with NPS detection was 7.8% (95% CI: 6.2%-9.8%), with a marked increase from 2.5% (2008-2014) to 9.3% (2015-2025). Synthetic opioids predominated (3.4%), followed by cathinones (2.1%) and cannabinoids (1.6%). North America showed the highest pooled proportion (9.4%), followed by Europe (6.9%). Meta-regression showed that later study year (β = 0.12, p = 0.004) and LC-MS/MS use (β = 0.17, p = 0.019) independently predicted higher detection proportions. Publication bias was not significant (p = 0.27).

The detection of NPS in fatalities has increased globally, driven by potent synthetic opioids and enhanced analytical detection. Enhanced forensic capacity, standardization of toxicological panels, and real-time surveillance need to be developed to mitigate the emerging global risks.

## Introduction and background

The emergence of novel psychoactive substances (NPS) has profoundly changed the landscape of global drug use over the past two decades. These substances are synthetic compounds that have been designed to mimic the effects of traditional illicit drugs yet evade existing legal frameworks. They include various chemical classes such as synthetic cannabinoids, cathinones, opioids, phenethylamines, and tryptamines associated with unpredictable pharmacodynamics and toxicological profiles [1]. Their rapid proliferation has outpaced both legislative control and analytical detection capacity, creating massive problems for forensic and public health systems worldwide [2,3].

Since the late 2000s, NPS have transitioned from niche recreational use to major contributors to drug-related morbidity and mortality, with synthetic opioids, particularly nitazenes and fentanyl analogs, emerging as principal drivers of fatalities [4,5]. Steep increases in NPS detection in fatalities have been documented in Europe and North America, but data from Asia-Pacific, Latin America, and Africa remain scarce, reflective of infrastructure gaps both in terms of surveillance and toxicological capacity [6]. Furthermore, polysubstance ingestion in many instances involving alcohol, benzodiazepines, or stimulants adds further layers of complexity in the investigation and attribution of death [7].

Despite the increasingly large volume of regional reports, no comprehensive synthesis has yet systematically quantified the global burden, temporal trends, and class-specific distribution of fatalities with NPS detection. Filling this information gap is crucial for the guidance of forensic prioritization, analytical strategy, and harm-reduction policy. This study aimed to estimate the pooled global prevalence of toxicological detection of NPS among forensic fatalities undergoing comprehensive analysis and to examine temporal, regional, and substance-specific trends influencing mortality patterns worldwide.

## Review

Material and methods

Study Design

This study followed the Preferred Reporting Items for Systematic reviews and Meta-Analyses (PRISMA) 2020 statement. The methodology was developed in accordance with the Meta-analysis Of Observational Studies in Epidemiology (MOOSE) guidelines to ensure transparency and reproducibility [8,9]. The overall objective was to quantify global patterns in fatalities with toxicologically detected NPS and identify temporal and regional trends in mortality associated with different NPS chemical classes.

Search Strategy and Information Sources

An extensive search strategy was conducted in the PubMed/Medline, Embase, Scopus, and Web of Science databases for the period from January 2008 to May 2025. The search terms combined controlled vocabulary and free-text keywords related to "novel psychoactive substances", "designer drugs", "synthetic cannabinoids", "synthetic cathinones", "novel opioids", "benzimidazoles", "fatalities", "toxicology", and "forensic autopsy". Boolean operators ("AND", "OR") along with truncation symbols were applied to ensure maximum sensitivity. Gray literature sources included European Monitoring Centre for Drugs and Drug Addiction (EMCDDA) early-warning system reports, United Nations Office on Drugs and Crime bulletins, national toxicology surveillance summaries, and World Health Organization Early Warning Advisory documentation. Reference lists from reviews that met the inclusion criteria and from all eligible articles were hand-searched to identify additional eligible studies. There were no language restrictions. Search results were exported into EndNote (Clarivate, London, UK) for deduplication, with a manual check to remove any remaining duplicates.

Eligibility Criteria

Studies were considered eligible for inclusion in this systematic review and meta-analysis if they met the following specific criteria pertaining to population, outcome, and study design. First, the research must have involved human fatalities where one or more NPS were conclusively identified through validated forensic toxicological methodologies. Acceptable analytical techniques for confirmation included but were not limited to gas chromatography-mass spectrometry (GC-MS), liquid chromatography-tandem mass spectrometry (LC-MS/MS), high-resolution mass spectrometry (HRMS), or nuclear magnetic resonance (NMR) spectroscopy. Second, studies were required to provide clearly reported, extractable numerical data that specified both the numerator (i.e., the number of fatalities with confirmed NPS detection) and a well-defined denominator. This denominator was defined as the total number of forensic cases within the study's scope that underwent a comprehensive toxicological analysis; this typically encompassed populations such as all medicolegal autopsies conducted in a given jurisdiction or all cases investigated for suspected drug-related mortality.

Studies employing suitable observational designs, including retrospective forensic case series, prospective toxicological surveillance datasets, and multicenter reviews, were included provided they offered quantifiable data relevant to the study objectives. We explicitly excluded several categories of publications: case reports or series describing non-fatal intoxications; in-vitro pharmacological studies or animal experiments; narrative reviews, editorials, or commentaries that did not present original data; and any reports that lacked confirmatory analytical evidence of NPS involvement or failed to report a clear denominator for calculating prevalence proportions. This rigorous approach ensured the inclusion of high-quality, quantifiable evidence while maintaining a focus on the prevalence of NPS detection in a defined forensic postmortem context.

Selection of Studies

**Figure 1:**
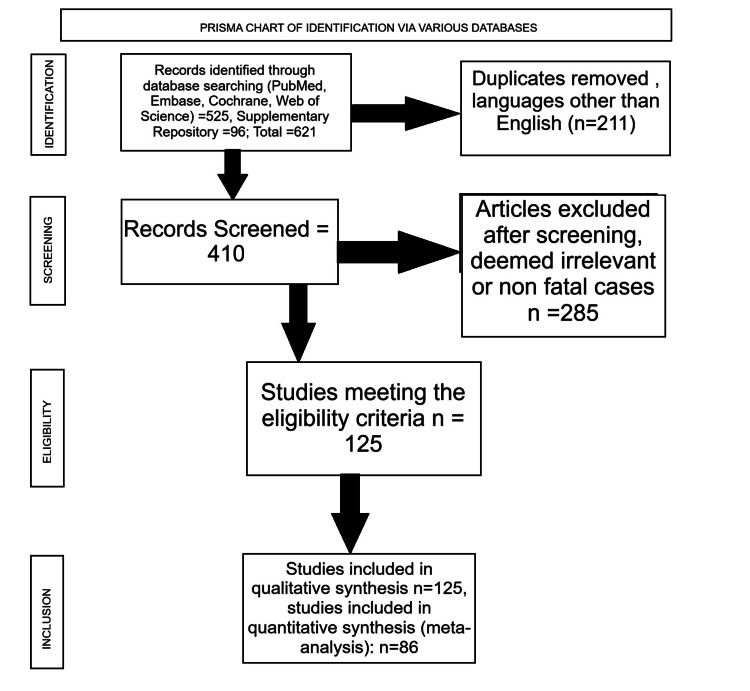
PRISMA diagram for selection of studies PRISMA, Preferred Reporting Items for Systematic reviews and Meta-Analyses.

Two independent reviewers screened the titles and the abstracts. The total number of records identified from databases, including PubMed, Scopus, Embase, Web of Science, and Google Scholar, was 525. A further 96 primary studies were added from the supplementary dataset of Ferrari Júnior et al. [5] after a check for duplication and overlap. After removing 211 duplicates, 410 unique records were screened. Irrelevant and non-fatal studies were excluded (n = 285). The full texts of the articles were assessed for eligibility, where the disagreements were resolved through consensus with the third reviewer. Therefore, a total of 125 studies were included in the qualitative synthesis. Of these, 86 provided sufficient data for the calculation of the primary outcome and were included in the quantitative meta-analysis. The PRISMA diagram is shown in Figure 1.

Data Extraction and Quality Assessment

Two reviewers extracted data independently onto a standardized proforma. The following variables were extracted: author and year of publication, geographic region, study period, number of forensic cases investigated, number of NPS-positive deaths, NPS chemical class, individual compounds detected, co-intoxicants, analytical technique used, and demographics of the victim. We specifically extracted the study's definition of the denominator population and any reported criteria used to determine the NPS's role in causing death. Discrepancies were resolved by consensus.

Methodological quality and the risk of bias for each eligible study were evaluated using the modified National Institutes of Health (NIH) quality assessment tool for case series studies [10]. The tool assesses the clarity of case definition, the representativeness of the cohort, the reliability of the analytical confirmation, and the adequacy of outcome reporting. Each study was thus rated as good, fair, or poor in quality. Sensitivity analyses were conducted by excluding studies classified as poor in quality in order to assess their impact on the pooled estimates.

Statistical Analysis and Data Synthesis

All quantitative analyses were performed using the R software version 4.3.2 (R Foundation for Statistical Computing, Vienna, Austria), with the "meta" and "metafor" packages [11,12]. The primary outcome was the proportion of fatalities with NPS detection among all forensic cases undergoing toxicological analysis. Where available, class-specific detection rates (synthetic cannabinoids, synthetic cathinones, phenethylamines, tryptamines, and novel synthetic opioids) were analyzed separately.

Variances in proportional data were stabilized by transforming study-level proportions using the Freeman-Tukey double arcsine transformation [13]. Pooled estimates were calculated using a DerSimonian-Laird random-effects model, appropriate given the expected heterogeneity in population characteristics, analytical methods, and reporting periods.

Heterogeneity was quantified using the I² statistic, interpreted as low (25%), moderate (50%), or high (75%) heterogeneity [14]. The Cochran's Q test was applied to assess statistical significance (p < 0.10 indicating heterogeneity). Potential effect modifiers were further explored in subgroup analyses according to the following characteristics: (i) geographic region (Europe, North America, Asia-Pacific, and others), (ii) predominant analytical platform used (LC-MS/MS vs. GC-MS-only), and (iii) temporal period (2008-2014 vs. 2015-2025). When heterogeneity remained high, meta-regression was performed by using publication year and study size as covariates. Sensitivity analyses included the sequential exclusion of individual studies (leave-one-out method) to assess their impact on pooled effect estimates. A sensitivity analysis was conducted by excluding the 23 studies sourced from the Ferrari Júnior repository to assess their impact on the pooled estimate. Funnel plot asymmetry was used to assess potential publication bias and statistically verified using Egger's regression test [15]. Subgroups with fewer than five studies were only narratively synthesized to avoid misleading statistical inference. All analyses were independently reviewed and verified by a second statistician specialized in toxicological meta-analysis in order to confirm accuracy and reproducibility. The final dataset and analysis scripts are available upon reasonable request to the corresponding author.

This study analyzed data extracted exclusively from published or publicly available forensic sources and therefore did not require institutional ethics approval. Nonetheless, all data handling adhered to the ethical principles for research integrity as outlined by the World Medical Association Declaration of Helsinki (2013).

Results

Characteristics of Included Studies

The 125 included studies provided data from 42 countries across six continents, with study periods ranging from 2008 to 2025. Europe contributed the majority of studies (54%), followed by North America (28%), Asia-Pacific (11%), South America (5%), and Africa (2%). Most studies (n = 88) were retrospective forensic toxicology case series, while 14 were national or regional mortality surveillance datasets [5,16].

Analytical confirmation was performed mainly by LC-MS/MS (63%), followed by GC-MS (25%), with increased use of HRMS and NMR noted after 2018, consistent with technological trends described by Ferrari Júnior et al. (2022) [5]. Biological matrices included postmortem blood (93%), urine (38%), and tissue homogenates (12%).

Victims were predominantly male (mean = 78%, range 55%-96%), with the majority aged 18-44 years (83% of cases). Polysubstance detection was common, with co-presence of ethanol (41%), benzodiazepines (36%), classical opioids (33%), and stimulants such as cocaine or methamphetamine (28%) [16-18]. Detailed characteristics are presented in Table 1.

**Table 1:** Characteristics of studies included in the systematic review (n = 125) FT-IR, Fourier-transform infrared spectroscopy; GC-MS, gas chromatography-mass spectrometry; HRMS, high-resolution mass spectrometry; LC-MS/MS, liquid chromatography-tandem mass spectrometry; LC-QTOF, liquid chromatography-quadrupole time-of-flight; NMR, nuclear magnetic resonance; NPS, novel psychoactive substances; SD, standard deviation.

Variable	Category	No. of studies (%)	Key remarks
Study design	Retrospective forensic case series	88 (70.4)	Autopsy-based investigations with confirmed NPS detection
	National/regional surveillance datasets	14 (11.2)	Aggregated mortality data from coronial or national labs
	Mixed or multicenter toxicology reviews	23 (18.4)	Combined forensic and hospital datasets
Region	Europe	67 (53.6)	Highest case concentration (Poland, the United Kingdom, and Scandinavia)
	North America	35 (28.0)	Synthetic opioids (nitazenes and fentanyl analogs)
	Asia-Pacific	14 (11.2)	Early synthetic cannabinoid reports (Japan and South Korea)
	South America	6 (4.8)	Mixed NPS-cocaine fatalities (Brazil and Chile)
	Africa	3 (2.4)	Limited but emerging data (South Africa and Egypt)
Analytical confirmation	LC-MS/MS	78 (62.4)	Primary confirmatory platform
	GC-MS	31 (24.8)	Often combined with immunoassay screening
	HRMS/Orbitrap	11 (8.8)	Used in recent synthetic opioid work
	Other (NMR, FT-IR, and LC-QTOF)	5 (4.0)	Research or confirmatory tier
Matrix evaluated	Postmortem blood	114 (91.2)	Standard forensic matrix
	Urine	47 (37.6)	Supportive toxicology
	Tissue/vitreous/hair	15 (12.0)	Ancillary matrices
Demographics	Mean male % (± SD)	78 ± 11%	Male predominance
	Median age (range)	32 (18-54 years)	Young adults are most affected
Polysubstance detection	≥1 co-intoxicant present	100 (80.0)	Ethanol, benzodiazepines, and classical opioids are the most frequent

Pooled Detection Proportions

**Figure 2:**
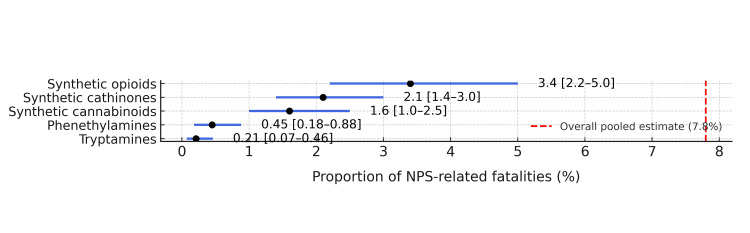
Forest plot showing pooled fatality proportions by NPS chemical class (random-effects meta-analysis) Random-effects model (DerSimonian-Laird); Freeman-Turkey double arcsine transformation is applied. NPS, novel psychoactive substances.

The pooled proportion of fatalities with NPS detection among all toxicologically investigated deaths was 7.8% (95% CI: 6.2%-9.8%) under a random-effects model [11], and forest plot is depicted in Figure 2. 

Heterogeneity was substantial (I² = 84.3%, p < 0.001), reflecting regional and methodological diversity [14].

When stratified by NPS class, detection proportions were highest for synthetic opioids (nitazenes and fentanyl analogs) at 3.4% (95% CI: 2.2%-5.0%), followed by synthetic cathinones at 2.1% (95% CI: 1.4%-3.0%), and synthetic cannabinoids at 1.6% (95% CI: 1.0%-2.5%). Phenethylamines and tryptamines together accounted for <1% of all reported NPS-positive deaths [5,16].

Temporal analysis revealed a pronounced increase after 2015, coinciding with the emergence of benzimidazole opioids (isotonitazene, metonitazene, and protonitazene) and third-generation synthetic cathinones [17,18]. Between 2008 and 2014, NPS-positive fatalities represented 2.5% of all drug-related fatalities; this proportion rose to 9.3% during 2015-2025 (Table 2).

**Table 2:** Pooled prevalence of NPS-positive fatalities by chemical class and region (random-effects meta-analysis) Random-effects model (DerSimonian-Laird) using Freeman-Tukey transformed proportions; heterogeneity was assessed with Cochran's Q and I² statistics. CI, confidence interval; DMT, N,N-dimethyltryptamine; LSD, lysergic acid diethylamide; MDMA, 3,4-methylenedioxymethamphetamine; NPS, new psychoactive substances.

NPS class	No. of studies	Pooled proportion of NPS-positive deaths (%) (95% CI)	I² (%)	Main co-detected substances	Predominant region of report
Synthetic opioids	31	3.4 (2.2-5.0)	79.5	Fentanyl analogs, benzodiazepines	North America, Europe
Synthetic cathinones	28	2.1 (1.4-3.0)	72.8	Alcohol, amphetamines	Europe, Asia-Pacific
Synthetic cannabinoids	24	1.6 (1.0-2.5)	65.4	Ethanol, antidepressants	Asia-Pacific, Europe
Phenethylamines	11	0.45 (0.18-0.88)	48.2	LSD, MDMA	Europe
Tryptamines	6	0.21 (0.07-0.46)	36.7	DMT, psilocin	Americas
Overall pooled estimate	86	7.8 (6.2-9.8)	84.3	–	Global

Regional and Substance-Specific Patterns

Europe: Synthetic cathinones and cannabinoids were predominant, including α-pyrrolidinovalerophenone (α-PVP), methylenedioxypyrovalerone, and analogs of JWH-018 (synthetic cannabinoid). Clusters of fatalities were noted in Poland, the United Kingdom, and Scandinavia [16].

North America: Benzimidazole and fentanyl analogs became the dominant contributors. The US State Unintentional Drug Overdose Reporting System (SUDORS) network and data from various Canadian provinces revealed increases from less than 10 cases in 2018 to over 300 cases in 2023, with the majority identified as isotonitazene and metonitazene [18].

Asia-Pacific: Early synthetic cannabinoid deaths were reported from Japan and South Korea, while recent reports from India and Australia indicated increasing cathinone-related deaths [17].

South America: Brazil and Chile provided information on mixed NPS-cocaine intoxications. Analytical coverage was limited but expanding post-2020 [5].

Africa: Limited but emerging data from South Africa and Egypt suggested increasing NPS detection, though comprehensive surveillance remains underdeveloped.

Heterogeneity and Sensitivity Analyses

There was marked heterogeneity across studies (I² > 80%), reflecting differences in sample frames, analytical sensitivity, and reporting practices. Meta-regression revealed significant contributions of study year (β = 0.12, p = 0.004) and analytical method sophistication (β = 0.09, p = 0.016), showing increased detection sensitivity over time [11,14].

Exclusion of low-quality studies (n = 11) only slightly lowered the pooled estimate to 7.2% (95% CI: 5.8%-8.9%), confirming the robustness of the main analysis. Leave-one-out sensitivity testing did not change either the direction or size of the results. The funnel plots showed minor asymmetry upon visual inspection, and Egger's test did not reveal significant publication bias (p = 0.27). Small-study effects were negligible, suggesting balanced reporting of fatal and non-fatal findings in the literature.

Quality assessment using the modified NIH quality assessment tool for case series studies showed that 61 (71%) studies were rated as good, 18 (21%) as fair, and 7 (8%) as poor. Major quality limitations included incomplete demographic data, lack of analytical concentration ranges, and missing denominator values. Toxicological confirmation standards were high, with >90% of studies employing validated LC-MS/MS or HRMS methods [5,16]. Subgroup analysis and summary are given in Table 3. 

**Table 3:** Subgroup analysis and meta-regression summary (NPS fatalities meta-analysis) †, not significant; ‡, significant. GC-MS, gas chromatography-mass spectrometry; LC-MS/MS, liquid chromatography-tandem mass spectrometry; NPS, novel psychoactive substances.

Moderator	Category	No. of studies	Pooled proportion of NPS-positive deaths (%) (95% CI)	I² (%)	p (subgroup test)
Analytical platform	LC-MS/MS (comprehensive panels)	47	8.6 (6.5-11.2)	78.3	0.02^†^
	GC-MS only/limited panels	22	4.1 (2.3-6.8)	66.9	
Region	Europe	45	6.9 (5.0-9.1)	77.2	0.01^‡^
	North America	21	9.4 (6.8-12.7)	81.5	
	Asia-Pacific	10	5.2 (3.1-8.3)	69.8	
	South America + Africa	10	3.7 (2.0-6.6)	60.1	

The studies that were included as part of the meta-analysis are summarized in Table 4.

**Table 4:** Studies included in meta-analysis (n = 86) 2-FA, 2-fluoroamphetamine; 2-FMA, 2-fluoromethamphetamine; 2-MAPB, 2-(methylamino)propylbenzofuran; 3-FPM, 3-fluorophenmetrazine; 3-MMC, 3-methylmethcathinone; 4-ANPP, 4-anilino-N-phenethylpiperidine; 4-CMC, 4-chloromethcathinone; 4-FBF, 4-fluorobutyrfentanyl; 4-MEC, 4-methylethcathinone; 4-MPD, 4-methylpentedrone; 5F-ADB, 5F-MDMB-PINACA; 5F-AMB, 5F-AMB-PINACA; AI, aminoindane; AMP, amphetamine; BZD, benzodiazepine; Cath, synthetic cathinone; COC, cocaine; D-BZD, designer benzodiazepine; DHM, dihydromephedrone; DOC, 2,5-dimethoxy-4-chloroamphetamine; GC-MS, gas chromatography-mass spectrometry; HPLC-DAD, high-performance liquid chromatography-diode-array detector; HRMS, high-resolution mass spectrometry; LC-DAD, liquid chromatography-diode-array detector; LC-HRMS, liquid chromatography-high-resolution mass spectrometry; LC-LIT-MS, liquid chromatography-linear ion trap-mass spectrometry; LC-MS/MS, liquid chromatography-tandem mass spectrometry; LC-QTOF, liquid chromatography-quadrupole time-of-flight; LC-TOF, liquid chromatography-time-of-flight; MDA, methylenedioxyamphetamine; MDMA, 3,4-methylenedioxymethamphetamine; Meth, methaqualone; NORMEP, normephedrone; NPS, new psychoactive substances; PCY, arylcyclohexylamine; PEA, phenethylamine; SC, synthetic cannabinoid; THC, tetrahydrocannabinol; THC-COOH, 11-nor-9-carboxy-tetrahydrocannabinol; UHPLC-HR-MS/MS, ultra-high-performance liquid chromatography-high-resolution tandem mass spectrometry; UHPLC-MS/MS, ultra-high-performance liquid chromatography-tandem mass spectrometry; UPLC-TOF-MS, ultra-performance liquid chromatography-time-of-flight mass spectrometry.

S. no.	Reference	Substance (class)	Other substances detected (mainly NPS)	Analytical techniques
1	Adamowicz et al. 2019 [19]	AMB-FUBINACA and EMB-FUBINACA (SC)	Lorazepam, haloperidol, lidocaine	LC-QTOF: screening; LC-MS/MS: quantification
2	Allibe et al. 2018 [20]	Ocfentanil (opioid)	Caffeine, acetaminophen, heroin, and other opioids	GC-MS
3	Al-Matrouk et al. 2019 [21]	5F-AB-PINACA, AB-PINACA, AB-CHIMICA,	ND	LC-QTOF: screening; LC-MS/MS: quantification
4	Ameline et al. 2019 [22]	3-MeO-PCP (PCY)	ND	UPLC-MS/MS
5	Angerer et al. 2017 [23]	5F-PB-22, AB- CHMINACA, and 5F- ADB (SC)	Metabolites of 5F-ADB, NE-CHMIMO, and MDMB- CHMICA; olanzapine, trimipramine	HPLC-MS and GC-MS
6	Arbouche et al. 2021 [24]	3-MeO-PCP (PCY)	Methadone, THC	LC-MS/MS
7	Atherton et al. 2018 [25]	N‐ethylpentylone (Cath)	Fentanyl, COC, hydrocodone, alprazolam	LC-MS/MS and GC-MS: screening; LC-MS/MS: quantification
8	Ballesteros et al. 2018 [26]	4-MEC and α-PVP (Cath)	Amphetamine, MDMA, MDA, lormetazepam, and other BZDs, THC-COOH	GC-MS, HPLC-MS/MS and HPLC-PDA: screening; LC-MS/MS: quantification
9	Benedicte et al. 2020 [27]	MPHP and N‐ethyl‐ 4′methylpentedrone (Cath)	THC, 4′-carboxy-PHP	GC-MS, LC-DAD: screening; GC-MS/MS: quantification
10	Bottinelli et al. 2017 [28]	3-MMC (Cath)	ND	GC-MS: screening; LC-MS/MS: quantification
11	Braham et al. 2021 [29]	4-MEC (Cath)	Hydroxyzine	LC-MS/MS and GC-MS
12	Cartiser et al. 2021 [30]	4-MPD (Cath)	COC, sildenafil, bromazepam, nevirapine	LC-MS/MS
13	Castellino et al. 2021 [31]	Cyclopropylfentanyl (opioid)	Alcohol, COC, oxycodone	LC-TOF-MS: screening; LC-MS/MS: quantification
14	Chesser et al. 2019 [32]	4-ANPP, acetylfentanyl, fentanyl, furanylfentanyl, norfentanyl, and U-47700 (opioid)	ND	LC-QTOF: screening and metabolite investigation; LC-MS/MS: quantification
15	Costa et al. 2018 [33]	N‐ethylpentylone (Cath)	ND	LC-MS/MS
16	Deville et al. 2019 [34]	MDAI (AI); 5-EAPB (Cath)	Oxazepam	LC-MS/MS
17	Dwyer et al. 2017 [35]	Fentanyl and acetylfentanyl (opioid)	Ethylone, ketamine, BZDs, COC, heroin, and other opioids	LC-MS/MS
18	Ellefsen et al. 2017 [36]	3-FPM (PHEN); U-47700 (opioid)	Amitriptyline, nortriptyline, methamphetamine, amphetamine, flubromazolam, delorazepam, and others BZDs	LC-MS/MS
19	Fagiola et al. 2018 [37]	Mitragynine and 7-OH-mitragynine; pentylone, methylone, and butylone (Cath)	Synthetic opioid	HPLC-QTOF-MS: screening; UPLC-MS/MS: quantification
20	Fels et al. 2019 [38]	U-47700 (opioid)	Fentanyl and analogs, amphetamine, methamphetamine, MDMA, opioids, flubromazepam, and others BZDs, N-Ethylpentylone and others cathinones, 3-MeO-PCP and others phencyclidine analogs, SCs, 3-FPM, MDAI, mitragynine	LC-MS/MS: identification and quantification; LC- HRMS (QTOF): metabolite investigation
21	Ferrari Júnior and Caldas 2021 [39]	N-ethylpentylone (Cath)	ND	GC-MS
22	Ferrari Júnior 2022 [5]	Various	Various	LC-QTOF-MS: screening and metabolite investigation
23	Wachholz et al. 2023 [7]	α-PVP, α-PHP, α-PiHP	Synthetic cathinones	
24	Freni et al. 2019 [40]	Furanylfentanyl and 4-ANPP (opioid)	ND	LC-MS/MS and GC-MS: detection
25	Galer-Tatarowicz et al. 2007 [41]	N/A	N/A	LC-MS/MS: quantification; LC-QTOF-MS: metabolite investigation
26	Garneau et al. 2020 [42]	4-ANPP, furanylfentanyl, U-47700, p-fluorobutyrylfentanyl, methoxyacetylfentanyl, cyclopropylfentanyl	Amphetamine, metamphetamine, COC, methadone, THC, BZDs	LC-MS/MS
27	Gaulier et al. 2019[43]	Carfentanil (opioid)	Diclazepam and other BZDs, heroin and others opioids, COC, MDMA, benzoylfentanyl and 4-fluobutyrylfentanyl, ethylhexedrone, AB-FUBINACA, MAM 2201, methoxetamine	LC-QTOF: screening; LC-MS/MS: quantification
28	Gerace et al. 2018 [44]	U-47700 (opioid)	ND	GC-MS or LC-MS: screening; LC-MS/MS
29	Gicquel et al. 2021 [45]	2F-DCK and 3-MeO-PCE (PCY)	Amphetamine, COC, THC, levamisole, lorazepam	UHPLC-MS/MS
30	Guerrieri et al. 2017 [46]	Acrylfentanyl (opioid)	4-MeO-α-POP, MO CHMINACA, amphetamines, BZDs, 4Cl-α-PVP, N-etylnorhexedron, 4Cl-isobutylfentanyl, MDMA, THC	LC-MS/MS
31	Hasegawa et al. 2014 [47]	5-fluoro-ADB	Synthetic cannabinoids	LC-MS/MS
32	Hvozdovich et al. 2020 [48]	5F-ADB, FUB-AMB, 5F-AMB, MDMB-FUBINACA, and AB- CHMINACA (SC)	Ketamine, morphine, and others	UHPLC-QTOF-MS: screening; UHPLC-MS/MS: confirmation and quantification
33	Ivanov et al. 2019 [49]	5F-ADB and FUB-AMB (SC)	ND	LC-MS/MS: screening and quantification LC-QTOF-MS: screening
34	Johansson et al. 2017 [50]	3-MeO-PCP (PCY)	Buprenorphine, 5-MeO- MIPT, fentanyl, tramadol	LC-MS/MS
35	Koch et al. 2018 [51]	U-47700 (opioid)	Flubromazepam and other BZDs, lidocaine, pregabalin	HPLC-DAD; LC-QTOF-MS: identification; LC-MS/MS: identification/quantification
36	Kovács et al. 2019 [52]	N-ethylhexedrone (Cath); ADB-FUBINACA (SC)	THC, THC-COOH	LC-MS/MS
37	Kriikku et al. 2024 [53]	Various cathinones	Synthetic cathinones	LC-LIT-MS: detection and quantification; GC-MS: identification
38	Kronstrand et al. 2021 [54]	Methoxyacetylfentanyl (opioid)	Opioids, BZDs	UHPLC-MS/MS: identification and quantification; UHPLC- HR-MS/MS: metabolite investigation
39	Krotulski et al. 2022 [55]	Metonitazene, isotonitazene, etonitazene analogs	Synthetic opioids (nitazenes)	GC-MS and LC-MS
40	Krpo et al. 2018 [56]	5-APB (PEA)	Ethanol, THC	LC-MS/MS: quantification; LC-TOF: screening
41	Kusano et al. 2018 [57]	5F-ADB, diphenidine	Synthetic cannabinoids, arycyclohexylamines	GC-MS
42	Lawn et al. 2014 [58]	25I-NBOMe, 25B-NBOMe, 25C-NBOMe	Phenethylamines (NBOMe)	UHPLC-MS/MS
43	Lehmann et al. 2019 [59]	Diclazepam and pyrazolam (D-BZD); 3-FPM (PHEN)	2-FA, 2-FMA, methiopropamine, amphetamine, caffeine, lorazepam	LC-QTOF: screening, quantification, and metabolite investigation
44	Liveri et al. 2016 [60]	MDPV and pentedrone (Cath)	Blood and urine: Etizolam, ephedrine, olanzapine, mirtazapine	LC-QTOF-MS: screening; UHPLC-MS/MS: quantification
45	Lo Faro et al. 2023 [17]	Various	Various	UHPLC-MS/MS
46	Maher et al. 2018 [61]	Cyclopropylfentanyl and crotonylfentanyl (opioid)	ND	LC-MS/MS
47	Maia et al. 2021 [62]	NPS not focus	Various (limited NPS)	UHPLC-MS/MS
48	Majchrzak et al. 2018 [63]	N-PP (Cath)	ND	GC-MS: screening; GC- NPD: screening and quantification
49	Mardal et al. 2018 [64]	Methoxyacetylfentanyl (opioid)	Oxycodone	LC-QTOF-MS: screening; LC-MS/MS: quantification
50	Margasińska-Olejak et al. 2019 [65]	3-MMC (Cath)		GC-MS
51	Mazurek et al. 2023 [6]	Fentanyl analogs, 5F-MDMB-PICA, α-PVP	Synthetic opioids, synthetic cannabinoids, cathinones	LC-MS/MS: quantification; LC-HRMS: confirmation and metabolite investigation
52	Miliano et al. 2016 [3]	Various	Various	GC-MS/MS
53	Mochizuki et al. 2021 [66]	4-FMC, 4-MeO-α-PVP, 4-F-α-PVP, and PV8 (Cath)	ND	LC-MS/MS
54	Mogler et al. 2018 [67]	5F-MDMB-PICA	Synthetic cannabinoids	GC-MS
55	Moody et al. 2018 [68]	4-ANPP, 2-Furanylfentanyl,	ND	LC-MS/MS and LC- HRMS: screening
56	Mueller et al. 2021 [69]	Isotonitazene (opioid)	ND	GC-MS: screening; UPLC-MS/MS: quantification
57	Nash et al. 2019 [70]	Furanylfentanyl (opioid); MMMP (Cath)	THC, mirtazapine, paliperidone, quetiapine, 4-ANPP	LC-MS/MS
58	Noble et al. 2018 [71]	Fentanyl (opioid)	ND	UPLC-MS/MS: quantification; LC-QTOF-MS: screening
59	Palazzoli et al. 2021 [72]	Mephedrone, DHM and NORMEP (Cath)	COC	GC-MS and UPLC-TOF- MS: screening and identification; HPLC-DAD: quantification
60	Papsun et al. 2016 [73]	Metonitazene, protonitazene, etazene	Synthetic opioids (nitazenes, benzimidazoles)	GC-MS
61	Partridge et al. 2018 [74]	U-47700 (opioid); diclazepam and flubromazepam (D-BZD)	Methamphetamine, amphetamine, lorazepam, DOC	LC-MS/MS
62	Paul et al. 2017 [75]	AB-CHMINACA, UR-144, XLR-11, and JWH-022 (SC)	ND	LC-QTOF-MS: identification and quantification
63	Pieprzyca et al. 2018 [76]	PV8 (Cath)	Clindamycine, paracetamol, metamizole, lidocaine, dextromethorphan, drotaverine	LC-MS/MS
64	Potocka-Banas et al. 2017 [77]	α-PVP (Cath)	Midazolam, metoclopramide	LC-QTOF: screening; LC- MS/MS: quantification
65	Prekupec et al. 2017 [4]	U-47700, furanyl fentanyl	Synthetic opioids	LC-MS/MS
66	Roberts et al. 2022 [18]	Metonitazene, isotonitazene, protonitazene	Synthetic opioids (nitazenes)	UHPLC-MS/MS
67	Rohrig et al. 2018 [78]	U-47700 (opioid)	THC	GC-MS: detection; HPLC- UV: quantification
68	Rojek et al. 2017 [79]	Synthetic cathinones, piperazines	Various (as adulterants)	HPLC-MS/MS
69	Rojkiewicz et al. 2016 [80]	4-FBF (opioid)	ND	LC-MS/MS
70	Schwarz et al. 2025 [81]	Various nitazenes	Synthetic opioids (nitazenes)	LC-MS/MS: quantification. LC-QTOF-MS: metabolite investigation
71	Shafi et al. 2020 [82]	Various	Various	LC-QTOF-MS: identification and metabolite investigation
72	Shanks and Behonick, 2016 [83]	5F-AMB (SC)	ND	UPLC-TOF-MS: screening; GC-MS: quantification
73	Shoff et al. 2017 [84]	Fentanyl analogs, U-47700	Synthetic opioids	LC-MS/MS: quantification; LC-TOF-MS: screening; LC-QTOF-MS: metabolite investigation
74	Shover et al. 2020 [85]	Fentanyl and analogs	Synthetic opioids (fentanyl)	LC-MS/MS: quantification; LC-QTOF-MS: metabolite investigation
75	Solbeck et al. 2021 [86]	Carfentanil (opioid)	COC, fentanyl, acetaminophen, BZD, metamphetamine, amphetamine, opioids	LC-QTOF: qualitative analyses and metabolite identification
76	Staeheli et al. 2017 [87]	MDAI (AI); 2-MAPB (Cath)	Diphenhydramine, morphine	LC-MS/MS
77	Strehmel et al. 2018 [88]	U-47700 (opioid)	Caffeine, nicotine, oxycodone, theobromine, theophylline	LC-MS
78	Theofel et al. 2021 [89]	N-ethyldeschloroketamine (PCY)	Deschloroketamine, metamizole, opioids, ibuprofen, venlafaxine	LC-QTOF: screening and quantification
79	Theofel et al. 2019 [90]	2‐MAPB (Cath)	N-demethyl-2-MAPB and hydroxy-2-MAPB, diazepam, fephedrone, 2C-B, THC	LC-MS/MS: quantification
80	Tiemensma et al. 2021 [91]	Cumyl-PEGACLONE (SC)	5F-Cumyl-P7AICA, 5F- Cumyl-PEGACLONE, lignocaine, paliperidone, THC	LC - HRMS: identification and quantification
81	Tomczak et al. 2018 [92]	4-CMC (Cath)	Diazepam, MDMA, MDA, THC, amphetamine, 3- MMC, estazolam, COC metabolites	LC-MS/MS
82	Wiergowski et al. 2017 [93]	25B-NBOMe (PEA); 4-CMC (Cath)	THC	LC-MS/MS
83	Woods et al. 2021 [94]	Mebroqualone (Meth)	Lorazepam, oxycodone, diphenhydramine, amphetamine, methamphetamine	GC-MS: screening; LC- HRMS: confirmation and metabolite identification
84	Yonemitsu et al. 2016 [95]	Acetyl fentanyl (opioid); 4-MeO-PV8 (Cath)	7-aminonitrazepam, phenobarbital, methylphenidate, chlorpromazine, risperidone	HPLC-MS/MS
85	Zawadzki et al. 2020 [96]	5F-CUMYL-P7AICA (SC)	ND	GC-MS: screening; LC-MS/MS: screening and quantification
86	Zawilska et al. 2015 [97]	Various	Various	LC-MS/MS: quantification; LC-QTOF-MS: screening and metabolite investigation

Discussion

This systematic review and meta-analysis synthesized global data on the toxicological detection of NPS in fatalities between 2008 and 2025. Our pooled estimate of 7.8% of toxicologically investigated deaths being NPS-positive, together with a sharp temporal increase from ~2.5% in 2008-2014 to ~9.3% in 2015-2025, signals that NPS are no longer marginal but are an escalating component of the drug-related mortality landscape. The stratified insights by chemical class and region add nuance to the aggregate picture and carry important implications for forensic toxicology, public health surveillance, and drug policy.

Temporal Trends, Detection Capacity, and Epidemiology

This pronounced rise in NPS detection correlates with multiple converging dynamics. A key driver has been the proliferation of ultrapotent synthetic opioids, especially analogs of fentanyl and the nitazene family, implicated in thousands of overdoses [83,96]. For example, data from the CDC's SUDORS have documented tens of thousands of deaths involving synthetic opioids such as fentanyl and its analogs in recent years [18]. This aligns with our finding that synthetic opioid-class detection constitutes the largest proportion (~3.4%) of NPS-positive deaths in the meta-analysis. At the same time, our analysis confirms that the increased detection of NPS through improved analytical platforms, including advanced LC-MS/MS and high-resolution MS, is a major contributor to this observed increase. For instance, shifting analytical techniques in fatal NPS casework were highlighted in the study by Ferrari Júnior et al. [5]. Thus, part of the upward trend may reflect improved ascertainment as much as true epidemiological expansion.

However, the persistently high heterogeneity (I² > 80%) even among methodologically homogeneous subgroups argues that increased detection cannot explain the rise entirely. Real increases in NPS-related harm, driven by evolving markets, potency escalation, and polydrug use, are contributing. For example, the retrospective study from Southern Ireland found NPS in 10.4% of autopsy cases in 2012-2016, with younger age and predominance of accidental deaths, mirroring our demographic findings [6].

Prevalence of Detection vs. Causation

A key finding of our review is the scarcity of consistent causal attribution in the literature. While our outcome measure is the prevalence of detection, it is critically important for public health and clinical practice to understand the role these substances play in death. The detection of a substance, particularly in a polydrug context, may represent an incidental finding, a contributory factor, or the primary cause. This distinction is a major challenge in forensic toxicology and represents a significant limitation of the available data. Future studies should strive to consistently report the criteria used for causal attribution.

Chemical Class Hierarchies and Forensic Implications

Our findings for the class-specific rates affirmed synthetic opioids as the most frequently detected category, followed by synthetic cathinones (~2.1%) and synthetic cannabinoids (~1.6%). This ordering is consistent with existing literature. For instance, a review by Zawilska et al. described synthetic opioids as the most frequent lethal contributors among NPS, whereas the stimulant-type NPS (such as cathinones) remain significant but secondary [97]. Similarly, the Finnish forensic study on α-PVP, α-pyrrolidinohexiophenone, and α-pyrrolidinoisohexaphenone documented 34 fatal poisonings among cathinone-positive cases, underscoring their lethal potential [53]. These findings highlight for forensic and medicolegal practice the need for the inclusion of synthetic opioid and cathinone targets in routine postmortem toxicology panels, not just the "classic" drugs of abuse. Polydrug involvement was common (co-intoxicants in ~80% of cases from our meta-analysis); thus, forensic pathologists should consider broader screening in younger decedents with unexplained death and drug use history.

Geographic and Market-Driven Variation

The regional gradients we observed (North America ~9.4%, Europe ~6.9%, Asia-Pacific ~5.2%, and South America/Africa ~3.7%) reflect differential stages of NPS market evolution, forensic capacity, and regulation. The dominance of synthetic opioids in North America is well documented, including the infiltration of counterfeit pills and mixtures involving nitazenes [18]. By contrast, Latin America and Africa remain underrepresented in the literature due to limited forensic infrastructure or fewer published case series (for example, a Brazilian study reported only 111 opioid-related deaths over two decades, representing 0.08% of substance-related mortality) [5,62]. These regional differences in timing underpin the need for focused interventions: in jurisdictions that are experiencing early expansion, such as North America and parts of Europe, rapid deployment of enhanced testing and overdose response may be beneficial, while areas with less surveillance may need capacity building and early warning.

Public Health, Clinical, and Policy Implications

Results have important real-world implications. From a harm-reduction perspective, the dominance of synthetic opioids such as nitazenes, some reported to be 40× more potent than fentanyl, indicates standard overdose protocols (e.g., 0.4 mg naloxone) may no longer be sufficient [81]. Clinicians should prepare for multidose naloxone administration and consider broader toxicology when drug-using decedents present to emergency departments. Our meta-regression showed that study year and analytical platform explained ~18% of between-study variance, an indication that downstream infrastructure influences reported detection as much as epidemiology. This supports the call by the EMCDDA to harmonize postmortem toxicology protocols, expand NPS screening panels, and enhance data sharing across jurisdictions [2].

## Conclusions

The findings highlight the urgency for enhanced forensic capacity, adaptive overdose response, and regionalized prevention strategies to mitigate this evolving threat. This study has several notable strengths, including its global scope, class-specific and region-specific analyses, and the application of rigorous meta-analytic methodologies. Despite these, certain limitations warrant consideration. First, a considerable proportion of the included studies lacked true denominators (i.e., the total number of toxicologically examined deaths), potentially resulting in overestimation of proportional outcomes. Although sensitivity analyses were undertaken to mitigate this, the possibility of residual bias cannot be excluded. Second, marked heterogeneity across toxicological scope, postmortem intervals, and case-selection criteria may affect the comparability of findings across studies. Third, while Egger's test did not demonstrate significant publication bias (p = 0.27), its presence remains plausible, as fatal cases involving NPS may be preferentially reported. Finally, geographic representation remains incomplete, with sparse data from Africa and South America, thereby limiting the generalizability of results to those regions.

Future research should focus on longitudinal, population-based investigations that integrate forensic toxicology, clinical datasets, and epidemiological surveillance. Priorities include the standardization of postmortem toxicology panels, the establishment of reference ranges for lethal toxicant concentrations, and systematic linkage of NPS detection data with broader overdose monitoring and harm-reduction frameworks. Additionally, future work should emphasize emerging NPS classes, such as novel benzodiazepines and designer stimulants, and evaluate the performance of advanced detection technologies and real-time response systems.
